# Factors related to advance directives completion among cancer patients: a systematic review

**DOI:** 10.1186/s12904-023-01327-w

**Published:** 2024-01-03

**Authors:** Mobina Golmohammadi, Abbas Ebadi, Hadis Ashrafizadeh, Maryam Rassouli, Salman Barasteh

**Affiliations:** 1 Student Research Committee, Baqiyatallah University of Medical Sciences, Tehran, Iran; 2 Behavioral Sciences Research Center, Life Style Institute, Baqiyatallah University of Medical Sciences, Tehran, Iran; 3 Nursing Faculty, Baqiyatallah University of Medical Sciences, Tehran, Iran; 4 Student Research Committee, Faculty of Nursing, Dezful University of Medical Sciences, Dezful, Iran; 5 Cancer Research Center, Shahid Beheshti University of Medical Sciences, Tehran, Iran; 6 Health Management Research Center, Baqiyatallah University of Medical Sciences, Tehran, Iran

**Keywords:** Advance care planning, Advance directives, Cancer, End-of-life preference, Nurses, Palliative care, Hospice, Decision-making

## Abstract

**Introduction:**

Advance directives (ADs) has recently been considered as an important component of palliative care for patients with advanced cancer and is a legally binding directive regarding a person’s future medical care. It is used when a person is unable to participate in the decision-making process about their own care. Therefore, the present systematic review investigated the factors related to ADs from the perspective of cancer patients.

**Methods:**

A systematic review study was searched in four scientific databases: PubMed, Medline, Scopus, Web of Science, and ProQuest using with related keywords and without date restrictions. The quality of the studies was assessed using the Hawker criterion. The research papers were analyzed as directed content analysis based on the theory of planned behavior.

**Results:**

Out of 5900 research papers found, 22 were included in the study. The perspectives of 9061 cancer patients were investigated, of whom 4347 were men and 4714 were women. The mean ± SD of the patients’ age was 62.04 ± 6.44. According to TPB, factors affecting ADs were categorized into four categories, including attitude, subjective norm, perceived behavioral control, and external factors affecting the model. The attitude category includes two subcategories: “Lack of knowledge of the ADs concept” and “Previous experience of the disease”, the subjective norm category includes three subcategories: “Social support and interaction with family”, “Respecting the patient’s wishes” and “EOL care choices”. Also, the category of perceived control behavior was categorized into two sub-categories: “Decision-making” and “Access to the healthcare system”, as well as external factors affecting the model, including “socio-demographic characteristics”.

**Conclusion:**

The studies indicate that attention to EOL care and the wishes of patients regarding receiving medical care and preservation of human dignity, the importance of facilitating open communication between patients and their families, and different perspectives on providing information, communicating bad news and making decisions require culturally sensitive approaches. Finally, the training of cancer care professionals in the palliative care practice, promoting the participation of health care professionals in ADs activities and creating an AD-positive attitude should be strongly encouraged.

## Introduction

Cancer is the main cause of death and an important barrier to increasing life expectancy in any country of the world. According to World Health Organization (WHO) estimates in 2019, cancer is the first or second cause of death before the age of 70 years in 112 out of 183 countries and the third or fourth in another 23 countries [1]. It is predicted that the number of cancer patients will reach from 9.96 million in 2020 to more than 16.3 million in 2040 [2]. Rapid deterioration and unexpected deaths occur in 22% of advance patients [3, 4]. Patients with advanced cancer and their caregivers often face severe physical, psychological, and financial consequences that compromise their quality of life (QOL) and/or end-of-life (EOL) quality. According to the principle(s) of palliative care (PC), one of the most important ethical and legal elements for PC is advance care planning (ACP) [5], which includes advance directives (ADs), health care agent and EOL medical decisions include do not resuscitate (DNR), and physician orders for life sustaining treatment (POLST) [6].

An AD as an important part of ACP is a written legal document in which a person can express wishes and preferences for medical treatment for the moment when that person is no longer able to make medical decisions because of a serious illness or injury. Therefore, an advance directive (AD) can preserve a person’s autonomy and self-determination once decision-making capacity is lost. In the last two decades of this century, many countries have approved principles of ADs and anticipated shared decision-making situations at the EOL by their legislative authorities [7]. An ADs provides a framework for patients to record thoughts about future medical care and treatment. It also ensures that if the person is unable to make decisions for themselves, they can follow the wishes and preferences recorded [8].

ADs have been shown to improve satisfaction with end-of-life care [9], reduce surrogate decision-making (SDM) conflict [10], and reduce hospital admissions [11]. Also, lack of ACP and/or ADs or their delayed implementation in cancer patients leads to higher in-hospital mortality [12, 13], greater use of resources near death [13–15], and delayed transfer to palliative or hospice care [12].

Despite the several benefits of completing ADs, studies have shown different rates of completing AD in different countries. For example, in the United States, a 2017 study found that approximately 1/3 of North Americans have completed AD [16, 17]. And one study in Australia showed only approximately 6% completion [18]. Access to AD is limited even in advanced cancer, with approximately less than half of cancer patients having documented AD [14, 19].

Studies have mentioned effective factors in the completion of effective ADs. In Chu study, AD completion was associated with patients aged ≥ 85 years and critical illness [20]. Alano et al. also mentioned the factors of gender, age, race, education, and religion as effective [21]. Del Pozo Puente et al. also showed education, lifestyle, chronic medication use, higher than average number of specialist visits, long-term relationship with family doctor, family history of Alzheimer’s disease and lower levels of social interaction [22]. Also, in cancer population older age, regular medication use, marital status, and permanent participation were factors that increased the likelihood of having an AD [23].

We used the theory of planned behavior (TPB) as a framework to elucidate the factors associated with ADs. The TPB states that “when an individual attempts to act, the intention to act is considered prior to performing the desired behavior, which is influenced by the individual’s attitude and subjective norms toward the behavior and sense of control over the behavior” [24]. An advantage of using the TPB for ACP implementation behaviour is that it can reveal the impact of subjective norms. Since previous studies have suggested that the values of people around an individual influence the implementation behaviour of ACP [22, 25].

To our knowledge, no systematic review has analyzed the factors influencing ACP from the perspective of patients based on using the TPB. However, others have systematically examined mental health service users’ perspectives [26] or barriers to the composition and implementation of ADs [27].

Although ADs can be helpful in maintaining the quality of a patient’s EOL [28, 29], the majority of people do not have an AD, mainly due to a lack of knowledge of ADs or because an AD is considered unnecessary now [30]. Consequently, the use of ADs in clinical practice remains low [31, 32]. Identifying and then reviewing the evidence for effective models for implementing and monitoring ACP interventions is needed [33].

Therefore, it is necessary to provide a standardized approach to integrating ADs into routine care for cancer patients to ensure that EOL medical care and treatment are aligned with the patient’s values, wishes, and goals. To achieve this goal, a clear understanding of the current ADs-related perspectives and factors should be provided. Therefore, the current systematic review and meta-synthesis study was conducted with the aim of determining the factors related to ADs from the perspective of cancer patients.

## Methods

### Study design

**Fig. 1:**
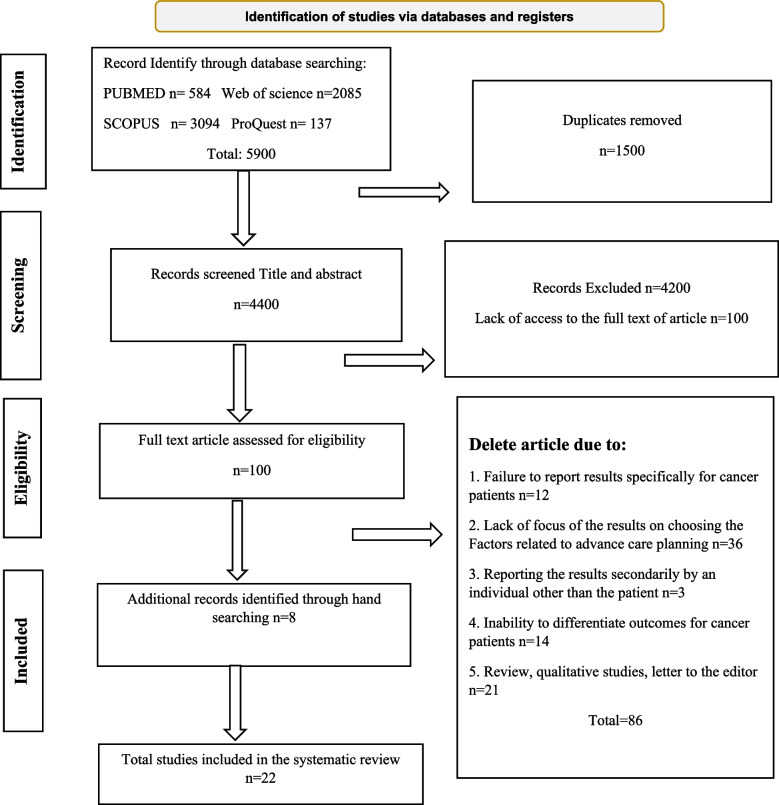
PRISMA flowchart

This systematic study was conducted based on the guidelines of preferred reporting items for systematic reviews and meta-analysis guidelines (PRISMA) [34] (Fig. 1). The study protocol is registered in PROSPERO with the code CRD42022301444.

### Type of study

All studies that explicitly stated the factors related to ADs from the perspective of cancer patients or provided data based on which the effective factors can be investigated were selected. Observational studies including cross-sectional descriptive study, semi-structured interviews, retrospective cohort, qualitative descriptive design, clinical trial and case control were included. Also, review article, case report, case series and clinical trial were not included in the present research.

### Type of participants

All primary studies, including case-control, cohort and cross-sectional studies, which were conducted on cancer patients of any race, ethnicity and one of the two gender groups, men or women or both genders and men, were included in the study.

### Sampling method & sample size

Sampling methods used in the studies included in the present systematic review are simple random sampling, systematic random sampling, stratified random sampling, cluster random sampling, quota sampling, convenience sampling, purposive sampling, self-selection sampling and snowball sampling) or primary studies that have used non-random (non-probability) sampling methods or announcing a public call or a combination of them.

### Exclusion criteria

Exclusion criteria include conference abstracts, case-report studies, reviews, gray studies and letter-to-the editor due to the lack of use of primary data, articles that have not provided a separate report on factors related to ADs from the perspective of cancer patients, studies focusing on pediatric cancer patients (people under 18 years old), studies focusing on factors related to ADs secondarily and tertiarily from nurses or physicians, doctors or family caregivers and the inability to separate outcomes for cancer patients.

### Search strategy

Studies were searched in four databases: PubMed/Medline, Scopus, web of science and ProQuest on November 8, 2021, without any time limit. To select keywords for this systematic review study, a combination of Mesh Term and Free Text words were used (Table 1. Search strategy). The search strategy was exactly the same in all databases. If there was no access to the full text of eligible articles, unpublished data or wrong and ambiguous data, an email was sent to the responsible author, and three more emails were sent at a 1–10-day interval. If no message is received from the article author after three emails, the article will be deleted. Any disagreement was resolved by the agreement of two researchers (S.B, M.G) and in case of disagreement; the decision was made based on opinion of the third informant.

**Table 1:** Search strategy

Search engines and databases: PubMed, Scopus, web of science, ProQuest
Limits: Language (Only resource with at least an abstract English)
Date: Up to 8 November 2021
(“Goals of care” OR ACP OR “Patient care planning” OR “Healthcare directive” OR “Health care directive” OR “Advance Directives” OR “Decision Making” OR “Patient Preference” OR “Personal Autonomy” OR “Advance care planning” OR “do not resuscitate order” OR “end of life discussions” OR “Anticipatory care plan” OR “future care planning” OR “Living Wills” OR “Resuscitation Orders” OR “Medical treatment order” OR “Statement of wishes” OR “Medical directive” OR “end of life discuss” OR “end of life conversation” OR “end of life decision” OR “end of life plan” OR “end of life preference” OR “advance medical plan” OR “advance statement”) AND TITLE cancer OR neoplasm OR tumor OR malignancy OR carcinoma

### Selection criteria

Original English articles published without time limit were identified according to the search criteria. Duplicate references were removed using EndNote X8. The titles and abstracts of the articles were checked in the screening stage. The selected studies were categorized into three categories: relevant, irrelevant and uncertain. Articles reported by both researchers as irrelevant were excluded from the study. Then, the full text of the articles was reviewed independently by two researchers in the selection stage (M.G. and S.B.). Any disagreement at any stage was resolved by discussion and agreement between the two researchers. In case of lack of consensus between the researchers, a third person was used as a referee and the result was reported in the form of statistical Kappa coefficient after reaching a general agreement. Afterwards, data extraction and quality assessment of the studies were carried out by two researchers (M.G. and S.B.).

### Risk of bias assessment

After investigating the objective of the studies and the inclusion criteria, 22 studies underwent quality assessment by two researchers (M.G. and S.B.) separately. Any disagreement between the two researchers was discussed and resolved. In case of lack of consensus between the researchers, a third person was used as a referee. The quality of these articles was evaluated using the Hawker et al. [35]. The reason for using this scale was the selection of qualitative, quantitative and mixed methods studies. Each study was evaluated on a scale from 4 to 1 (4: good, 3: fair, 2: poor, and 1: very poor). Each study was assessed in a scale ranging from 4 to 1 (4: good, 3: fair, 2: poor, and 1: very poor) (Details of the assessment are shown in Table 2).

**Table 2:** Assessment of articles included in the review

**Authors/ Year**	**Abstract and title**	**Introduction and aims**	**Method and data**	**Sampling**	**Data analysis**	**Ethics and bias**	**Results**	**Transferability generalizability**	**Implications usefulness**	**Total**	**Average**	**Grade**
Wang et al. 2021 [36]	4	4	3	3	4	4	4	3	4	33	3.6	Good
Cohen et al. 2021 [37]	3	3	3	3	3	3	4	3	2	27	3	Good
Berkowitz et al. 2021 [38]	4	4	3	4	4	3	3	4	3	32	3.5	Good
Bar-Sela et al. 2021 [39]	3	3	3	3	3	4	4	4	4	31	3.4	Good
Rodenbach et al. 2020 [40]	4	4	3	3	4	4	4	4	4	34	3.7	Good
Brown et al. 2016 [19]	4	4	4	3	3	3	4	3	4	32	3.5	Good
Kish et al. 2000 [41]	2	3	4	3	3	3	3	3	3	27	3	Good
Dow et al. 2010 [42]	4	4	2	2	4	3	2	3	3	27	3	Good
Kubi et al. 2020 [43]	4	4	3	3	3	3	4	4	4	32	3.5	Good
Saeed et al. 2019 [44]	4	3	3	4	3	3	3	3	3	29	3.2	Good
Prater et al. 2019 [45]	4	4	4	4	3	3	4	3	3	32	3.5	Good
Bires et al. 2018 [46]	3	3	4	2	3	3	3	3	4	27	3	Good
McDonald et al. 2017 [31]	4	4	2	3	3	3	3	4	4	30	3.3	Good
Kim et al. 2017 [47]	4	4	3	2	3	3	3	4	4	30	3.3	Good
Zheng et al. 2016 [13]	4	4	3	3	4	3	4	4	4	29	3.2	Good
Tan and Jatoiet al. 2008 [48]	4	4	4	4	3	3	4	4	4	34	3.7	Good
True et al. 2005 [49]	4	3	3	3	4	3	4	3	4	31	3.4	Good
Seifart et al. 2020 [50]	4	4	3	3	4	3	4	3	3	31	3.4	Good
Zaros et al. 2013 [51]	3	3	3	2	3	3	4	3	3	27	3	Good
Wallace et al. 2001 [52]	4	4	3	3	3	3	4	4	4	32	3.5	Good
Lin et al. 2019 [53]	4	4	3	2	3	3	3	3	3	28	3.1	Good
Sudore et al. 2018 [54]	4	4	4	4	4	4	4	4	4	36	4	Good
Total	3.7	3.6	3.1	3	3.3	3.1	3.5	3.4	3.5	30.2	3	Good

To preserve the data, studies with a score lower than the average score (less than score 3) were considered to have a poor quality. None of the 22 studies were excluded due to poor quality.

### Data extraction

First, a data extraction form was developed and the factors affecting ADs were analyzed by two researchers (M.G. S.B.) separately using the directed content analysis approach [55]. The use of models and theories of behavior change helps to identify the characteristics of people and the surrounding environment that influence their behavior [56]. In the present study, the TPB constructs was used to investigate the factors affecting ADs from the perspective of patients with advanced cancer. According to this model, the most important determinant of a person’s behavior is his intention to perform the behavior. Behavioral intention is influenced by three variables: attitude, subjective norms and perceived behavioral control [56]. First, full texts of the articles were read several time to get an overview. Then the factors affecting ADs were extracted as meaning units and codes. At first, an article was evaluated as a pilot using this form; then the same process was carried out for other articles. Any disagreement between the researchers was resolved through discussion and agreement. In case of lack of consensus between the researchers, a third person was used as a referee and the result was reported after reaching a general agreement. The specifications of the articles including the author, year of study, location of study, sample size, study design, factors related to ADs and study quality were extracted. The factors related to ADs were extracted from the perspective of cancer patients using this form, and the results of the studies were categorized in the form of different factors.

## Results

After searching for keywords in databases, a total of 5900 articles were found. A total of 4400 articles remained after removing duplicate references. Afterwards, 4293 articles were excluded due to lack of inclusion criteria. The remaining 108 articles were reviewed in detail based on their full text. Finally, 22 eligible articles were included in the study (Details of the studies included in the review presented in Table 3).

**Table 3:** Characteristics of the studies included in the review

**Authors Year**	**Aim**	**Country**	**Methodology**	**Sample and setting**
Wang et al. 2021 [36]	To describe the knowledge and preferences of ADs and EOL care decisions of patients with brain tumors	China	cross-sectional	Patients from Cancer Hospital, Chinese 316 Brain Tumor Patients
Cohen et al. 2021 [37]	To investigate of the potential association between ACP and hope in advanced cancer	USA	cross-sectional	672 patients with cancer from 17 medical oncology practices within the University of Pittsburgh Medical Center Hillman Cancer
Berkowitz et al. 2021 [38]	To investigate of components of ACP in patients with cancer as compared with patients with noncancer serious illness referred to palliative care	USA	cross-sectional	1,604 patients with cancer and 1,094 patients without cancer in Duke University Medical Center
Bar-Sela et al. 2021 [39]	To investigate of evaluate the barriers and motives among Israeli cancer patients regarding ACP	Israel	mixed-methods	109 Israeli advanced cancer patients from the Division of Oncology at Rambam
Rodenbach et al. 2020 [40]	To investigate of relationships between patients’ worry about dying and their illness understanding, treatment preferences, and ACP	USA	cross-sectional	672 patients with solid tumors at 17 cancer clinics in western Pennsylvania
Brown et al. 2016 [19]	To evaluate patients’ knowledge regarding ADs	USA	prospective study	110 gynecologic cancer patients from Anderson Gynecologic Oncology Clinic in Houston
Kish et al. 2000 [41]	To investigate of describe cancer patients admitted to an ICU with and without ADs	USA	prospective study	872 patients with Malignancy in Anderson Cancer Center
Dow et al. 2010 [42]	To investigate of determine with which of their physicians patients preferred to discuss ADs	USA	Qualitative/ Semi-structured interviews	75 patients with cancer in Virginia Commonwealth University Medical Center Hematology
Kubi et al. 2020 [43]	To investigate of oncology patients’ preferences surrounding ACP with a focus on the choice of which health care providers to have the conversation with and the timing of conversations	USA	cross-sectional	200 oncology patients from the surgical oncology and medical oncology units of Johns Hopkins Hospital
Saeed et al. 2019 [44]	investigate of effect of income and education on the completion of ADs	USA	cross-sectional	256 patients were had stage IV nonhematologic cancer or stage III cancer in the Rochester/Buffalo, New York, and Sacramento, California regions
Prater et al. 2019 [45]	investigate of determine the impact of advance care planning (ACPEOL) among a sample of hospice-referred patients with cancer	USA	retrospective cohort	1185 patients with a primary diagnosis of cancer from a Midwestern academic medical center
Bires et al. 2018 [46]	Investigate of understand the various challenges and personal beliefs regarding ACP through interviews with two groups: patients undergoing active cancer treatment and their oncology providers	USA	Qualitative/ semi-structured interview	10 oncology providers and 20 patients from 39 to 69 years in multispecialty, ambulatory care cancer center in the Atlantic region
McDonald et al. 2017 [31]	Investigate of awareness and prevalence of ADs among patients with advanced cancer undergoing active outpatient care and to determine factors associated with AD completion before and after the diagnosis of cancer	Canada	cross-sectional	395 patients with advanced solid tumor malignancy in Cancer Centre, University Health Network, Toronto
Kim et al. 2017 [47]	Investigate of undertaken to examine the extent to which cancer patient–caregiver dyads would utilize the K–AD and their level of agreement about EoL decisions	Korean	descriptive study	81 cancer patients in from one of two tertiary hospitals in Korea
Zheng et al. 2016 [13]	To investigate knowledge and attitudes of approving ADs and explore factors associated with willing to designate ADs among cancer patients in China	China	Qualitative/ semi-structured interview	753 in-patients with cancer in Two departments of oncology from two university hospitals
Tan and Jatoi et al. 2008 [48]	To assess current rates of ADs among patients with incurable pancreas cancer	USA	retrospective study	1,186 consecutive patients with unresectable pancreas cancer in Mayo Clinic in Rochester/Minnesota
True et al. 2005 [49]	Investigated of differences between African American and White patients with cancer in their use of spirituality to cope with their cancer and examined the role of spiritual coping in preferences at EOL	USA	study group	68 patients with an advanced stage of lung or colon cancer from the Albert Einstein Cancer Center
Seifart et al. 2020 [50]	Investigation of gender differences concerning the content, the desired time point, and the mode of initiation of EOL conversations in cancer patients	German	cross-sectional	186 female and male cancer patients in University Hospital Marburg
Zaros et al. 2013 [51]	To examine physician assessment of decisional capacity and the prevalence of EOL discussions during the terminal hospitalization of patients with advanced cancer	USA	retrospective cohort	145 cancer patients in the University of Michigan Hospital
Wallace et al. 2001 [52]	Investigation of the presence of an AD at admission to an intensive care unit (ICU) influenced the decision to initiate life support therapy in critically ill cancer patients	USA	case-control	872 patients treated in the Anderson Cancer Center ICU
Lin et al. 2019 [53]	Investigation of decision-making processes and drivers of receiving palliative care in ACP discussions from perspectives of advanced cancer patients, families and healthcare professionals in northern Taiwan	Taiwan	Qualitative/ Semi-structured interviews	45participants oncology unit and one hospice unit in a tertiary hospital
Sudore et al. 2018 [54]	Engaging Diverse English- and Spanish-Speaking Older Adults in Advance Care Planning The PREPARE Randomized Clinical Trial	USA	Clinical trial	986 English-speaking and Spanish-speaking older adults with chronic illness from 4 primary care clinics

It should be noted that only articles related to the perspective of cancer patients towards ADs were reviewed in the present study, and other studies were excluded. In total, the views of 9061 cancer patients were examined, among whom 4714 were women and 4347 were men. The mean ± SD of people’s age was 62.04 ± 6.44. Most of the articles were published to the United States included (*n* = 15 cases) [19, 37, 38, 40–46, 48, 49, 51, 52, 54], followed by China (*n* = 2 cases) [13, 36], and South Korea (*n* = 1) [47], Canada (*n* = 1) [31], Israel (*n* = 1) [39], Germany (*n* = 1) [50] and Taiwan (*n* = 1) [53].

Factors related to ADs were extracted based on the model of planned behavior in four main categories, including attitude, subjective norms, perceived control behavior and external factors affecting the model (Table 4).

**Table 4:** Categories and subcategories extracted from studies

**Categories**	**Subcategories**
Attitude	Lack of understanding of the ADs concept
	Previous experience of the disease
Subjective norms	Social support and interaction with family
	Respecting the wishes of the patient
	EOL care choices
Perceived control behavior	Decision making
	Access to the healthcare system
External factors affecting the model	Socio-demographic characteristics

### Attitude

Attitude in TPB refers to a person’s positive or negative evaluation of a specific behavior, which depends on the person’s judgment about the effects and consequences of the behavior [56]. The attitude category included “Lack of knowledge of the ADs concept” and “Previous experience of the disease”.

#### Lack of knowledge of the ADs concept

Knowing and familiarizing with the ADs concept is an effective factor in the willingness of patients to complete the AD form. In this regard, in the study in China, Wang et al. referred to knowledge and awareness about AD as the most important factor in having it from the patients’ perspective [36]. On the other hand, Bar-Sela et al. [39] stated most of the cancer patients had not heard anything about AD, and as a result, they did not want to write it due to insufficient knowledge of ACP services. Also, McDonald et al. in their study in Canada reported the lack of knowledge of the AD concept as the most important barrier in completing AD in patients [31]. Zheng et al. also referred in their study in China to patients’ lack of information about AD as a barrier to completing it [13]. Similarly, Kim et al. stated that lack of information about AD caused patients to not complete it [47]. Cancer patients who did not have enough information about their disease [46, 47] stated that AD was not necessary and there was no advantage to think about writing it [42]. They felt that thinking about their treatment priorities makes them sad and depressed [44] and causes disruption in the work process of treatment and care services [39].

#### Previous experience of the disease

Previous experience of the disease can indirectly affect the completion of AD. In this regard, Kish et al. found that patients with hematologic malignancies completed AD more frequently than patients with solid tumors, and patients with recurrent or progressive cancer also completed AD more frequently than patients in remission [41]. Also, Cohen et al. and Berkowitz et al. reported that patients who were less affected by cancer were less likely to complete the AD form [37, 38]. Patients with a history of functional impairments and cognitive impairment preferred supportive treatments over invasive treatments and were less likely to have living will (LW) [41].

### Subjective norms

Subjective norms in TPB refers to the social pressure estimated by a person to perform the desired behavior or not [57]. Factors related to subjective norms included “social support and interaction with family”, “respecting patient’s wishes” and “EOL care choices”.

#### Social support and interaction with family

Social support and interaction with family as the first subcategory of subjective norms can be an effective factor for having AD. In this systematic review study, social support means the level of interaction and intimacy and effective communication of the patient with family and relatives and the support received from the health care system [58].

In a study by Bar-Sela et al., the main motivation of patients to have AD is to talk with family and treatment team and express their care priorities so that patients sought an opportunity to discuss with family and treatment team to complete their ACP process [39]. In their study, Wang et al. also expressed that patients complete AD in order to establish extensive communication with family and health care providers and concluded that patients want to have effective communication and interaction with their family and treatment team to avoid unnecessary treatments and avoid being a burden on the family [36]. McDonald et al. also referred to the support-counseling system of palliative care after cancer diagnosis as one of the factors related to the completion of AD by patients [31]. On the other hand, a previous study by Kubi et al. in the United States referred to the patient’s distrust of the hospital and the treatment staff, as well as the lack of trust in the family as motivations for completing AD [43]. Other factors affecting AD included patients’ belief that they have no one to provide them with EOL care [36, 39, 43], the lack of support and encouragement from the family to write AD [36, 39], as well as the lack of extensive communication and interaction with the family to express treatment preferences due to fear and worry of death [19, 40] and the reaction of family members [47, 59] were.

#### Respecting the patient’s wishes

Respecting the patient’s wishes was the second sub-category. In Kubi et al.’s study, patients believed that they should write the AD as soon as possible in order to respect their wishes and prevent others from imposing their contradictory opinions on them [43]. In their study, Lin et al. referred to the respect for patients’ wishes for treatment decisions as the right of choice for the patient and respect for his/her rights [53]. A total of 66% of patients in McDonald et al.’s study [31] had written their EOL preferences and wishes and had completed the AD form.

#### EOL care choices

Based on the results obtained from the included studies, the use of hospice services [47], PC, DNR [45], were the most important EOL priorities of the patients. Supportive treatments were chosen more frequently than invasive treatment when patients talked about EOL priorities and completed the AD form [41]. However, in Kim S et al.’s study, 44.4% of participants referred to difficulty to choose EOL care and frustration after AD registration as reasons for their failure to complete the AD form [47].

### Perceived control behavior

Perceived behavioral control refers to the degree to which a person believes that he or she can perform a given behavior [57]. “decisions making”, “participating in life activities” and “access to the healthcare system” were the three subcategories of perceived behavior control.

#### Decision making

Decision-making is carried out in two general ways in the included studies, i.e. by the individual [43] and with the help of others including family and healthcare service providers, which is mentioned in four studies [36, 37, 51, 53]. According to the results of Kubi et al.’s study, patients stated that their opinions regarding care priorities may be inconsistent with family members and relatives; therefore, it is better to decide on having ACP as soon as possible [43]. On the other hand, in Bar-sela et al.’s study, a group of patients with lung, pancreas, stomach and brain cancers believed that they trusted a family member who had been appointed as a durable power of attorney (DPOA) to make decisions for them when needed [39]. The patient completes the DPOA form in order to make a decision for him in case of incapacity [60]. Finally, the patients stated that they fully trust their spouse and children or the doctor and social worker to make the best medical decision for them [39]. In the study by Wang et al., 30.91% of patients believed that healthcare service providers help them when they need them, and 13.64% of them also believed that the family makes decisions instead of them [36]. 84.8% of patients in Cohen et al.’s study preferred to choose a surrogate decision maker (SDM) to choose their care priorities [37] and also 95% of patients in Lin et al.’s study chose their spouse as a SDM [53]. According to the study by Zaros et al. and Wallace et al. one of the challenges of patients with a SDM compared to those without a SDM was more invasive treatments such as mechanical ventilation, chemotherapy, nasogastric tube feeding, and longer ICU stay [51, 52].

#### Access to the healthcare system

Various factors predicted the completion of the AD form in the subgroup of access to the healthcare system. For example, the role of health insurance that covers the highest level of medical services was referred to in the study by Wang et al. [36]. Also, the place to receive cancer care and treatment was expressed by Tan and Jatoi as a motivation to complete the AD form [48]. Cohen et al. also referred to the role of time of receiving care from healthcare providers 1 to 6 months after cancer diagnosis as a motivation to complete the AD form [37]. In McDonald et al.’s study and Bar-Sela et al.’s study, the quality of care and access to PC services and healthcare providers were referred to as factors affecting patients’ willingness to complete the AD form [31, 39].

### External factors affecting the model

The last category, external factors affecting the model included “socio-demographic characteristics”. In the current study, socio-demographic characteristics including age, economic status and income level, level of education, marriage, religious beliefs and gender were effective in completing the AD form by patients.

There is a direct relationship between patients’ age and completion of the AD form, so that the older they become, the more inclined they were to complete the AD form, which is consistent with studies by Tan and Jatoi [48], McDonald et al. [31], Prater et al. [45], Saeed et al. [44], Bar-Sela et al. [39] and Brown et al. [19]. The level of income and economic status, is considered as an effective factor for completing the AD form in the studies by McDonald et al. [31], Cohen et al. [37]. That is, the higher the income level, the more inclined patients were to complete the AD form. Saeed et al. [44], also stated that patients with lower income level were less likely to complete the AD form.

In this systematic review study, patients with high school diploma and college level of education agreed to complete AD more than patients with lower educational level, which is consistent with studies by Cohen et al. [37], Lin et al. [53], Brown et al. [19], and Kubi et al. [43].

Most patients who agreed to complete the AD form were married [31, 37, 39, 42, 53]. There is only one study in the USA that stated being single is as an effective factor in completing the AD form for patients [48].

Wang et al., and Kubi et al. found that men were more likely to complete the AD form [36, 43]. In contrast, Zheng et al. found that the AD form was completed by women more frequently than men [13]; But it was mentioned in two studies that gender was not an effective factor in completing the AD form AD [47, 50]. True et al. stated that patients who believed in the power of God and spirituality to cope with cancer were less likely to have LW [49]. In a study by Wang et al. in China, 2.72% of patients with brain tumors referred to religious beliefs as a barrier to completing the AD form [36]. In contrast, religious beliefs were regarded as an effective factor to complete the AD form in studies by Zheng et al. [13] and Cohen et al. [37].

## Discussion

According to the results of the present study, ADs -related factors were categorized in the four main categories based on TPB, including attitudes, subjective norms, perceived control behavior, and external factors affecting the model.

The attitude was recognized as the first category of ADs-related factors, which included the two sub-categories of lack of knowledge of the ADs concept and previous experience of the disease. The results of the past studies in this area show that the patient’s knowledge and attitude towards EOL patient care guidelines and the disease prognosis is very important in this area [13, 36, 39, 47]. After being introduced with the ACP concept, patients with difficult to cure diseases prefer to complete the AD form [36]. The ACP is a process of conversation, discussion, and an official document between patients and their caregivers about their demands for treatment and care planning when they are unable to make medical decisions due to illness or disability. In several countries, AD has been become a law since the California Natural Death Act in 1976 [61–63]. Patient Self-Determination ACT (PSDA) requires that Medicare and Medicaid providers notify all patients of their rights to complete the AD form to determine their health care priorities [64]. The biggest barrier to completing the AD form is that some people consider AD a similar act like passive ethanasia since it denies sustainable treatments for patients with incurable diseases. However, in practice, family members often abandon sustainable treatments for a patient with incurable diseases [65]. EOL issues such as ACP, PC, death place and family roles are valuable for cancer patients [66, 67]. Discussing EOL preferences with patients with cancers and a variety of malignancies is very important. Effective conversation ensures that patients have accurate opinions on EOL preferences, such as treatments or prognosis [68]. Healthcare providers must advertise all EOL guidelines to allow patients make the right decisions. The results of some included studies show that EOL patients were willing to discuss ACP, but in fact, their healthcare providers rarely discussed this with their patients. This may be due to the fact that medical staff refused to raise this issue because they are worried that early ACP set up can cause fear in patients or create a feeling of despair regarding their prognosis [69]. However, the above evidence shows that providing appropriate education to healthcare providers and ACP information to patients [70] actually helps facilitate ACP discussion between patients and their healthcare providers, and thus promotes ACP exercises.

The previous experience of the disease was another effective predictor of ADs. Participants with longer duration of disease, less passive coping styles, more active coping styles, and previous experience of taking care of a dying family member will have a higher preparedness for ACP [71], which is consistent with previous studies by Loberiza et al. [72]. A person with active coping strategies generally solves stress problems by trying to change or eliminate stressors [73]. In contrast, when people feel difficult to control a situation, passive coping style is used and tend to protect their emotional state that is first affected by stressful events. However, it should be noted that people can use both active and passive coping strategies to manage their stress at the same time [74]. To increase the ACP preparedness, healthcare professionals can encourage patients to seize their active independence in EOL decisions and provide emotional management to patients to reduce their psychological burden [71]. Besides, patients with a longer disease duration may experience disease recurrence and opportunities to consider the disease prognosis more frequently. The study population and caregivers in the Fried et al.’s study [75] stated that fear of bad experience during death increased their participation in ACP. Therefore, it is suggested that the previous EOL caregivers experience can encourage patients to think about death and thus enhance the ACP preparedness.

The category included the subjective norms that consisted of three subcategories of “social support and interaction with family”, “respecting the patient’s wishes” and “EOL care choices”.

Social support and interaction with the family were the first subcategory of the subjective norms. There have been numerous studies on the protective effects of high-quality social relationships on healthy behaviors, so that People with close and supportive relationships are more likely to have more health-promoting behaviors than people with poor relationships [76]. According to the main themes of social support views, warm and supportive relationships may increase one’s general willingness to participate in ACP and help them appoint a family member as DPAHC with whom he/she has a high-quality relationship. People who report higher levels of family general performance and frequent emotional support from their spouses and children are more likely to be involved in ACP than those with lower-quality relationships [77]. According to social control themes, people with high-quality relationships have the incentive to participate in ACP because their spouse or child encourages them to do so [78] or because they know that this ACP may help protect their loved ones against difficult EOL decisions [79]. Recent studies on social control perspectives suggest that the effectiveness of social control efforts may mainly depend on the quality of one’s relationships with the control agent [80]. Overall, positive interaction and tactics, such as support and encouragement, are more effective than negative cases such as reprimand or harassment. Since ACP is considered as a preventive health behavior and can ultimately predict the experience of a better death for the patient [9], there has been a great deal of emphasis on the availability of warm and supportive relationships elsewhere to reduce distress and promote healthy behaviors [76].

Respecting for the patient’s wishes by focusing on the patient’s individual independence is considered an important factor in ADs. In general, the ACP process means expanding individual independence in the face of inability to make decision. One of the ACP goals states “ACP is generally supported as a tool whereby patients can expand the participation and control of their health care decisions beyond the point where they have lost their capacity due to illness or damage [69]. Considering this basic goal, ACP confirms that health care decisions should be based on patient preferences and treatment will match these preferences that ultimately increases the sense of self-control in patients [81]. Critics emphasize that preferences may change over time or with the disease progression. ACP is thought to overcome many of these problems because it is a continuous process for defining, revising and documenting preferences. ACP might also improve the applicability of ADs, by specifying how the AD is to be used. ACP must clarify whether decisions are made by family members or only by one person, and is understood as the leeway when interpreting preferences” [82]. Therefore, patients’ preferences and values should be evaluated regularly. To reflect patient preferences and goals, training programs are essential for medical specialists that facilitate specialized knowledge and skills about ACP and EOL care.

After reviewing the literature on EOL priorities, the results of some included study show that ACP activities are potentially effective tools to support EOL care based on patients ‘preferences and values. EOL includes eliciting and respecting the patient’s priority for providing high-quality care [83]. ACP and advanced instructions are critical tools achieving such goal [84]. A key concern is that the patients’ EOL preferences change over time and their ACP or AD documents are not updated regularly, then the preference recorded months or years ago may no longer valid in the EOL decisions [85]. The results of some included articles show that people’s EOL preferences vary between ethnic groups. In this regard, the results of a systematic review show that the EOL priority was fixed for more than 70% of patients [86]. Among patients with advanced cancer who prioritized comfort-focused care at the EOL, engaging in an EOL discussion with their physician and completing DNR orders were both significantly related with receiving valuable EOL care [87].

Perceived control behavior as the third category related with ADs consisted of two subcategories of “Decision making” and “Access to the healthcare system”. After a complete review of the included studies, the results of the present study show that EOL decision-making is carried out either by the person alone or relatives, including the family and healthcare providers. Despite advances in preventive, early diagnostic measures, and new treatments, many patients reach end-stage cancer, which leads to situations where decision-making is difficult for patients, caregivers, and physicians [88]. There are some clinical and technical criteria to guide EOL decisions, but the condition of the dying patient is so closely related to specific personal circumstances that decisions are largely guided by clinical judgment and prior expertise [89]. If a patient not only wishes to have their wishes contribute to clinical decision-making, but to actually make his/her own decisions in advance to refuse certain treatments, the ACP process should include supporting patients to complete an advance decision to refuse treatment as a binding legal precedent for those decisions [90] Silveira et al. showed that many cancer patients need to make decisions when they lack the capacity to do so. Besides, those patients who had ADs or had appointed a durable power of attorney received more consistent EOL care preferences. Silveira et al. further mention that 92.7% of patients with ADs preferred not to receive invasive EOL care, while 1.9% wanted to receive all possible care [28]. Meanwhile, a concept called shared decision-making provides an approach to discuss ACP in a collaborative and informed manner. Shared decision making is defined as a process in which the patient and healthcare professionals make decisions together using the best available evidence [91]. Shared decision making incorporates the principles of patient-centered care, which seeks to provide high-quality care by acknowledging patients’ personalities in all aspects of care, and is, therefore, relevant in ACP [92]. Furthermore, shared decision-making models typically define decision-making as a process that provides patient health care [93]. However, evidence suggests that caregivers will be involved in EOL decisions in up to 78% of cases [94]. Therefore, EOL decision-making models should consider the distinct roles of patients, caregivers, and healthcare providers [95]. Also, primary tools used to document ADs include a durable health care power of attorney to designate a substitute decision maker and a “living will” such as cardiopulmonary resuscitation, mechanical ventilation, and the use of medical hydration and nutrition in some cases, which usually addresses individual preferences for sustainable treatments [96].

The access to the healthcare system in the present study included various concepts such as the role of health insurance, the place of receiving and providing healthcare services, the time of receiving healthcare services, the quality of care, and access to palliative care services and health care providers. A person’s decision to use healthcare services is affected by a complex interaction of factors related to a person’s health and self-reported health status and the availability of healthcare services. The results of some studies show that the lack of governance and management policy to create a supportive culture for EOL care at the organizational level negatively affects the working environment that is conducive to interdisciplinary teamwork and thus hinders the implementation of ACP [97].

Finally, as the last category that was extracted from the results of the studies included external influential factors in the form of socio-demographic variables. Individual socio-demographic variables and health status have been shown to be related to the acceptance and completion of the AD form in the general population. Previous studies reported a direct relationship between AD complementation with older age [20, 21, 98], female gender [21, 98], higher education [21, 30, 99] and higher income [99]. Older cancer patients usually talk about death more than younger patients, and most of them wish a death with dignity, but are not sure how to die, so, they are willing to use AD to put their wishes on paper; thus, they create less psychological burden for their family members and friends [13]. Besides, a relationship was reported between the AD completion rate with poorer health status, critical illness or chronic medication use [20].

Also, the summary of studies shows that religious patients are more likely to complete the AD form, which is similar to previous studies showing a significant relationship between religion and completion of the AD form [100, 101]. Overall, patients confirm religion as an important consideration in their lives [102]. Religious participants indicated that the manner of death and self-medical decision should be consistent with their religious teachings and values. They often discussed their disease and treatment plan during hospitalization and preferred comfort or limited care near EOL because of their faith. However, the results of some studies show that healthcare providers rarely pay attention to their religious beliefs [13]. The reasons for the contradiction between the results of studies regarding the relationship between demographic variables and completion of AD can be due to different personalities, different populations and different sample sizes. Andersen’s expanded behavioral model of health service use has been widely used as a conceptual framework to explain differences in the use of various types of health care, including EOL care. This model assumes that predisposing factors (e.g., age, sex, marital status, level of education), factors related to perceived need (e.g., chronic conditions, functional disability) and empowering factors (facilitators and barriers of health services including income, health insurance, socio-economic status, acculturation and characteristics of the health system) which were mentioned in the previous paragraph, greatly affect the utilization of healthcare services [103–105].

## Limitation

One of the limitations of the present study was that it only considered English language studies. Another limitation was patients with different stages of the course of cancer and their disease conditions and cancer types were different. This may affect the findings, so conducting more studies in cancer patients in a specific phase of the disease and also investigating the factors related to ADs from the perspective of other chronic patients and in different age groups and comparing they are important with cancer patients. The next limitation is that most of the included studies were conducted in Western countries with different religions and it may not be possible to generalize it to East Asian countries. It is recommended that other studies be conducted with the aim of examining the views of patients in different age groups, with different physical and mental conditions in Western countries and East Asia and compare the results.

Furthermore, because this review included a variety of studies with diverse designs, direct comparison of effectiveness between results was difficult.

## Conclusion

The results of the current study have classified the ADs- related factors into four main categories based on the model of planned behavior, including attitudes, subjective norms, perceived control behavior and external factors affecting the model. The attitude category consisted of “lack of knowledge of the ADs” and “previous experience of the disease”. The subjective norm included three subcategories of “social support and interaction with the family”, “respecting for the patient’s wishes” and “EOL care choices”. The perceived control behavior also consisted of two subcategories of “decision making” and “Access to the healthcare system”. Finally, factors related to socio-demographic variables are emphasized as external factors. Therefore, to perform ADs, policy makers should consider the importance of facilitating open communication between patients and their families and different perspectives on information delivery, communication of bad news and decision-making, culturally sensitive approaches. ADs completion should focus on exploring patients’ values, rather than making treatment plans in advance. Identifying barriers that limit ADs completion and primary palliative care referrals can certainly help prioritize next steps for future studies that aim to promote the culture of ADs completion and help physicians better support patients shared decision-making based on the patient’s values and experiences. Finally, the training of cancer care professionals in the practice of PC, promoting the participation of health care professionals in ADs and creating a positive attitude towards the ADs should be strongly encouraged.

## Implications for policymaking and education

Additional training in the field of ACP and ADs is recommended for health professionals so that they are familiar with the latest information and related tools. Education and appropriate data tools for promotion of ADs completion culture are important as they may decrease reluctance and promote ADs use. Also, policy makers should develop culturally acceptable ADs guidelines as well as develop ADs policies and legal frameworks. This paper contributes to the wider global policymakers by pointing out the importance of standardizing ADs contents and practices.

## Data Availability

All data generated or analyzed during this study are included in this published article.
